# Low-value care practice in headache: a Spanish mixed methods research study

**DOI:** 10.1186/s10194-020-01147-w

**Published:** 2020-06-10

**Authors:** Patricia Pozo-Rosich, Almudena Layos-Romero, Jimmy Martin-Delgado, Julio Pascual, Cristina Bailón, Ana Tentor, Alejandro Santiago, Emilio Ignacio, Antonio Torrés, José Joaquín Mira

**Affiliations:** 1 Headache Unit, Neurology Department, Vall d’Hebron Hospital, Barcelona, Spain; 2 Headache and Neurological Pain Research Group, VHIR, Universitat Autonoma of Barcelona, Barcelona, Spain; 3 Albacete General University Hospital, Albacete, Spain; 4 ATENEA research group, Foundation for the Promotion of Health and Biomedical Research, Sant Joan d’Alacant, Spain; 5 University Hospital Marqués de Valdecilla, Santander, Spain; 6 University of Cantabria and IDIVAL, Santander, Spain; 7 Barrio del Pilar Health Center, Madrid, Spain; 8 University Hospital Infanta Leonor, Madrid, Spain; 9 Cádiz University, Cádiz, Spain; 10 Patient Safety Observatory, Andalusian Healthcare Quality Agency, Seville, Spain; 11 Health Department Sant Joan d’Alicante, Alicante, Spain; 12 Miguel Hernández University of Elche, Elche, Spain

**Keywords:** Care, Cost, Do not Do recommendations, Headache, Patient safety, Mixed methods

## Abstract

**Background:**

Headache is one of the most prevalent diseases. The Global Burden of Disease Study ranks it as the seventh most common disease overall and the second largest neurological cause of disability in the world. The “Do Not Do” recommendations are a strategy for increasing the quality of care and reducing the cost of care for headache. This study aimed to identify specific low-value practices in headache care, determine their frequency, and estimate the cost overrun that they represent, in order to establish “Do not Do” recommendations specifically for headache by consensus and according to scientific evidence.

**Methods:**

This was a mixed methods research study that combined qualitative consensus-building techniques, involving a multidisciplinary panel of experts to define the “Do Not Do” recommendations in headache care, and a retrospective observational study with review of a randomized set of patient records from the past 6 months in four hospitals, to quantify the frequency of these “Do Not Do” practices. We calculated the sum of direct costs of medical consultations, medicines, and unnecessary diagnostic tests.

**Results:**

Seven “Do Not Do” recommendations were established for headache. In total, 3507 medical records were randomly reviewed. Low-value practices had a highly variable occurrence, depending on the hospital and type of headache. Overall, 34.1% of low-value practices were related to treatment, 21% were related to overuse of imaging in consultation, and 19% were related to emergency care. The estimated cost of low-value practices in the four hospitals was 203,520.47 euros per 1000 patients.

**Conclusions:**

This study identified low-value headache practices that need to be eradicated and provided data on their frequency and cost overruns.

## Introduction

Headache is one of the most prevalent diseases and one of the main reasons for consultation in primary care and neurology services [1]. The prevalence of headache is around 51% and, in Spain, it has been estimated that more than four million people suffer from migraine. Of these, one million suffer from chronic migraine [2, 3]. The Global Burden of Disease Survey [4] ranked headache as the seventh most common disorder causing the greatest decrease in quality of life in years. When considering only neurological diseases, migraine is the second most common disabling condition, after stroke. According to the World Health Organization (WHO), headaches are one of the most disabling health problems with the highest socioeconomic impact, since they are responsible for the inactivity of sufferers during the most productive years of their lives [5, 6].

The Global Campaign against Headache, led by Lifting the Burden, together with the WHO, aims to reduce the gap in organizational, diagnostic, and therapeutic approaches to headache worldwide, and particularly in Europe, and seeks to identify the quality dimensions of headache care that should be considered in order to provide integrated quality care [7, 8]. The Eurolight study analyzed the organization and treatment of patients with migraine in Europe and estimated that the annual cost of care for headache patients is 173,000 million euros [9]. Migraine accounts for 64% of this total cost (110,000 million euros). Spain ranks second in Europe in terms of the direct annual cost of headache [10]. The 2018 Atlas of Migraine in Spain estimated that the annual total direct cost per patient for healthcare services was 3847.29 euros for chronic migraine and 964.19 euros for episodic migraine. To these amounts must be added the cost assumed by the patient, which ranges from 1657.96 euros for chronic migraine and 878.04 euros for episodic migraine; and the indirect cost associated with the inability to work for this reason, which has been estimated at 7464.83 euros for chronic migraine and 3199.15 euros for episodic migraine. The total cost per patient per year comes to 12,970.08 euros for chronic migraine and 5041.38 euros for episodic migraine [11].

Overuse is defined as “the provision of medical services for which the potential for harm exceeds the potential for benefit” [12]. Ultimately, overuse can be considered to occur along a continuum. At one end of the continuum lie tests and treatments that are universally beneficial when used for the appropriate patient. At the other end of the continuum are services that are entirely ineffective, futile, or that possess such a high risk of harm to all patients that they should never be delivered. Most procedures and interventions lie between these extremes, and consensus is needed to help elucidate whether these procedures and interventions should be recommended, and to help decide which patient they may benefit [13].

The “Less is More,” “Right Care,” “Choosing Wisely,” or “Do Not Do Recommendations” campaigns have sought to establish consensus criteria to identify practices that have low value in health care, in an effort to reduce them. Locally, the “Commitment for the Quality of Care of the Scientific Societies in Spain,” of which 49 scientific societies form part, have elaborated a list of recommendations, each one in its specialty or specific field, about what should not be done following the methodology of “Choosing Wisely.” In this case, there is only one recommendation for headache care in Spain (Do not Do neuroimaging studies repeatedly in patients with primary headache (migraine and tension headache) without changes in their profile [14]). Although scientific societies internationally have established consensus regarding the overuse of imaging tests or treatments that should be avoided, there is still room to justify the importance of reaching agreements regarding other practices that do not add value to specific headache care.

In this study, we aimed to identify specific low-value practices in headache care, determine their frequency, and estimate the cost overrun that they represent. “Do not Do” recommendations specifically for headache were established by consensus and according to scientific evidence.

## Methods

This was a mixed methods research study that combined qualitative consensus-seeking techniques, involving a multidisciplinary panel of experts, to define “Do not Do” practices in headache, and a retrospective observational study, with review of a random set of medical records from four hospitals to quantify the frequency of these “Do not Do” practices. This study was conducted between June and December 2019.

### Review of the literature

To prepare for the working session with the panel of experts, a narrative review of the literature was carried out after searching the following databases: PubMed, Scopus, and EMBASE. The following search terms were used: “headache,” “migraine,” “neuroimaging,” “Global Campaign Against Headache,” “quality of care,” “do not do,” “guidelines,” “treatment. Additionally, a manual search was carried out on the websites of the following organizations: Sociedad Española de Neurología, Atlas de Migraña, Choosing Wisely USA, Choosing Wisely Canada, Choosing Wisely Australia, American Headache Society, International Headache Society, National Institute for Health and Care, and Scottish Intercollegiate Guidelines Network (SIGN).

The titles and summary of the studies identified were reviewed by two authors to decide whether they were relevant to this study. The full text of the selected articles or documents was revised to extract information on “Do not Do” recommendations in headache. The information was included based on its level of evidence, and feasibility of execution. It was agreed that the SIGN classification should be used as a guide to determine the levels of evidence and degrees of recommendation. Prior to the working session of the group of experts, the information was organized according to the stage of the care process, for example, diagnosis, evolution, treatment, or control, and whether it was an emergency care situation or a neurological consultation.

### Group working session

The group of experts consisted of three specialists in neurology, and one specialist each in family medicine, hospital pharmacy, and neurological nursing. The selection of these panelists took into account the involvement of professionals throughout the care process, as well as their experience from the first level of care to monographic consultations for headache, as well as their specialized training. The work of the panel of experts included a group session and multiple telematic meetings until a sufficient degree of consensus was reached. Experts had gathered information during the literature review as well as additional evidence that they could provide. In the face-to-face meeting of the panel of experts, the recommendations identified in the literature review were analyzed one-by-one. In parallel, experts proposed and debated “Do not Do” recommendations associated with the headache care process in their areas of expertise. JM and JJM produced a synthesis of the debate and recorded recommendations through summary sheets that were analyzed individually by successive rounds of telematic meetings, and thereby “Do not Do” recommendations were identified. The consensus on which “Do not Do” recommendations should be proposed considered the quality of the evidence for each recommendation, the feasibility in identifying whether the undesirable practice was implemented in clinical practice, and the foreseeable acceptance of the recommendation by the professional community. Summary cards included the code of the “Do not Do” recommendation, its definition, current problem, number of instances of the undesirable practice occurring within a given number of consultations, sources of information, and evidence.

### Field study

We measured the frequency by which these low-value or undesirable practices were implemented in an observational, retrospective, multicenter study. In this study, 3507 clinical records of patients who attended four university hospitals of the Spanish public health system over the past 6 months were randomly reviewed. A minimum required sample size of 3068 patients was calculated for a precision of 3%, *p* = q = 50% worst-case scenario, an expected proportion of losses due to incomplete information of 15%, and a confidence level (two-tailed) of 95%. The hospitals had between 367 and 1146 beds. The review of clinical records was carried out by applying algorithms for data extraction from clinical information systems that defined each of the “Do not Do” recommendations determined by consensus in the first part of the study. The International Classification of Diseases (ICD-10) and the Anatomical Therapeutic Chemical Classification System (ATC) were used to select patients who had attended the hospital for headache.

### Direct costs

Direct costs of ignoring this Do not Do recommendations were estimated by summing the estimated costs of medical consultations, diagnostic tests, and medicines associated with low-value practices. This estimate was made considering the average costs of consultations and diagnostic tests in the health services of Madrid, Castile and Leon, and Murcia (based on data from 2017) and the reference prices for financing medicines by the National Health System in Spain described in Order SCB/1244/2018. The average cost of the first consultations was 82 euros, successive consultations were 71 euros, and emergency care was 175 euros. Brain functional studies with magnetic resonance imaging had an average cost of 400 euros, and electroencephalogram 147 euros.

The overall direct cost of these low-value practices at each center in the study period (6 months) was obtained by multiplying the direct cost, in euros, of consultations, tests, and medicines by the volume of patients with low-value practices. Finally, considering a prevalence of 12–13% for migraine, 2–3% for chronic migraine, and 51% for headache [11], we extrapolated the total direct cost for the Spanish healthcare system for the agreed low-value indications, by multiplying the cost per patient by the number of patients that could be expected to have low-value indications according to our data.

## Results

The literature review identified nine relevant studies and three technical papers. Fourteen “Do not Do” recommendations and two proposals for discussion by the expert group were developed from this information. Overall, the expert panel suggested 17 “Do not Do” recommendations at the group work session (supplementary file is available with all the recommendations proposed by the expert panel). Finally, seven “Do not Do” recommendations were prioritized through telematic means based on the quality of the evidence for each recommendation, the feasibility in identifying whether the undesirable practice was implemented in clinical practice (data extraction from the clinical information systems contributes to this identification), and the foreseeable acceptance of the recommendation by the professional community. Full details of these seven “Do not Do” recommendations are available in Table 1.

**Table 1:** “Do Not Do” Recommendations in headache. Results of the consensus search panel

Code	Definition	Evidence
NHC-01	Do not recommend painkillers (NSAIDs, paracetamol, and others) more than 15 days per month in a primary headache that does not respond to treatment. Numerator: Number of patients with primary headache (ICD-10-ES G44.2, G44.53, G44.83, G44.84 and G44.85) who have continuous pharmacological treatment of more than 15 days with NSAIDs/acetaminophen. ATC GROUPS: ergotics: N02CA02, N02CA52; acetaminophen: N02BE01; AAS: N02BA01; NSAIDs: M01A Denominator: Total number of patients treated for primary headache.	Diener et al. Pathophysiology, prevention, and treatment of medication overuse headache. Lancet Neurol. 2019;18(9):891–902. Diener et al. Chronic headache due to overuse of analgesics and anti-migraine agents. Dtsch Arztebl Int. 2018;115:365–370. Becker et al. Medication overuse headache in Canada. Cephalalgia. 2008;28(11):1218–20. Cheung et al. Medication overuse headache. Curr Neurol Neurosci Rep. 2015;15(1):509. Headache Classification Committee of the International Headache Society (IHS). The International Classification of Headache Disorders, 3rd edition (beta version). Cephalalgia. 2013;33(9):629–808.
NHC-02	Do not withdraw preventive treatment in patients with primary headaches before 8 weeks if the efficacy and therapeutic dose is adequate. Numerator: Number of patients with preventive treatment ATC GROUPS Beta blockers: C07AA05, C07AA12, C07AB03, C07AB02; Antiepileptics: N03AX11, N03AG01; Renin-angiotensin inhibiting drugs: C09AA03, C09CA06; N07CA03; Antidepressants: N06AA09, N06AX16; Botulinum Toxin: M03AX01 not meeting criteria Denominator: Total patients being treated for primary migraine headache with (G43.1) and without aura (G43.0), chronic migraine (G43.3) and cluster headache (G44.0)	SIGN 155. Pharmacological management of migraine. A national clinical guideline. February 2018 Hepp et al. Persistence and switching patterns of oral migraine prophylactic medications among patients with chronic migraine: A retrospective claims analysis. Cephalalgia 2017,37(5):470–485.
NHC-03	Do not perform neurophysiological studies to establish the diagnosis of migraine or tension headache. Numerator: Number of patients who have undergone neurophysiological studies (ICD-10-ES 4A00) Denominator: Total number of patients under treatment for primary migraine headache with (G43.1) and without aura (G43.0), chronic migraine (G43.3), cluster headache (G44.0) and tension headache (G44.2).	Guías diagnósticas y terapéuticas de la Sociedad Española de Neurología 2015;3. Guía oficial de práctica clínica en cefaleas. Editores: P Pozo Rosich, D Ezpeleta. Luzán5. Madrid 2015. ISBN: 978–84–15,198-99-4. Gronseth GS, Greenberg MK. The utility of the electroencephalogram in the evaluation of patients presenting with headache: a review of the literature. Neurology 1995;45(7):1263–1267.
NHC-04	Do not perform neuroimaging studies on patients with tension headache, migraine without aura, and those with typical and episodic aura without alarm signs. Numerator: Number of neuroimages (CIE-10-ES BW28, BW38) performed in patients with the diagnoses of Tension Headache (G44.2), Migraine without aura (G43.00), and Migraine with aura (G43.1) and Headache (R51) Denominator: Total number of neuroimages performed in the service per headache (CIE-10-ES R51)	Becker et al. Guideline for primary care management of headache in adults. Can Fam Physician. 2015;61(8):670–679. Ezpeleta D, Pozo-Rosich P, eds. Guía oficial de práctica clínica en cefaleas de la Sociedad Española de Neurología 2015; ediciones SEN. Guía práctica diagnóstico terapéutica de la cefalea del adulto y el niño en urgencias 2016. Guías diagnóstico-terapéuticas de la Sociedad Española de Neurología. Editores: A Macaya, P Pozo-Rosich. Luzán5. Madrid 2016. ISBN: 978–84–7989-867-0.
NHC-05	Do not refer patients from primary care with a diagnosis of primary headache without having prescribed symptomatic treatment with triptans or standard preventive treatment. Numerator: Number of patients referred without preventive treatment guidelines Beta-blockers: C07AA05, C07AA12, C07AB03, C07AB02; Antiepileptics: N03AX11, N03AG01; Renin-angiotensin inhibitor drugs: C09AA03, C09CA06; N07CA03; Antidepressants: N06AA09, N06AX16; Botulinum toxin: M03AX01 not met. Denominator: Diagnosis of migraine without aura, migraine with aura, and others not specified (ICD-10-ES G43.00, G43.10, G43.70, G43.90).	Guías diagnósticas y terapéuticas de la Sociedad Española de Neurología 2015. 3. Guía oficial de práctica clínica en cefaleas. Editores: P Pozo Rosich, D Ezpeleta. Luzán5. Madrid 2015. ISBN: 978–84–15,198-99-4. Gago-Veiga et al. How and when to refer patients diagnosed with primary headache and craniofacial neuralgia in the emergency department or primary care: recommendations of the Spanish Society of Neurology’s Study Group. Neurologia 2017. pii: S0213–4853(17)30268–2.
NHC-06	Do not perform complementary neuroimaging tests on an urgent basis on patients who do not meet the established alarm criteria. Numerator: Number of neuroimages (ICD-10-ES BW28, BW38) performed on patients in the emergency department who do not meet any alarm criteria Denominator: Total number of neuroimages performed on patients with primary headache: migraine with (G43.1) and without aura (G43.0), chronic migraine (G43.3) and cluster headache (G44.0) in the emergency department.	Expert panel on Neurologic Imaging. ACR Appropriateness Criteria: Headache, revised 2019. Do et al. Red and orange flags for secondary headaches in clinical practice: SNNOOP10 list. Neurology. 2019;92(3):134–144. Guía práctica diagnóstico terapéutica de la cefalea del adulto y el niño en urgencias 2016. Guías diagnóstico-terapéuticas de la Sociedad Española de Neurología. Editores: A Macaya, P Pozo-Rosich. Luzán5. Madrid 2016. ISBN: 978–84–7989-867-0.
NHC-07	Do not use opiates as a symptomatic treatment for primary headache. Numerator: Number of patients with opioid indication ATC GROUPS (N02AA) Denominator: Total number of patients evaluated in the service for primary migraine headache with (G43.1) and without aura (G43.0), chronic migraine (G43.3) and cluster headache (G44.0).	Guías diagnósticas y terapéuticas de la Sociedad Española de Neurología 2015. Guía oficial de práctica clínica en cefaleas. Editores: P Pozo Rosich, D Ezpeleta. Luzán5. Madrid 2015. ISBN: 978–84–15,198-99-4. Guía práctica diagnóstico terapéutica de la cefalea del adulto y el niño en urgencias 2016. Guías diagnóstico-terapéuticas de la Sociedad Española de Neurología. Editores: A Macaya, P Pozo-Rosich. Luzán5. Madrid 2016. ISBN: 978–84–7989-867-0. Becker WJ. Acute migraine treatment in adults. Headache. 2015;55(6):778–793. Evers S, Afra J, Frese A, Goadsby PJ, Linde M, May A, Sandor PS, European Federation of Neurological Societies. EFNS guideline on the drug treatment of migraine-revised report of an EFNS task force. Eur J Neurol. 2009;16(9):968–981.

NHC: “Do not Do” in headache (translation from Spanish “No Hacer en Cefalea”)

*ATC* Anatomical Therapeutic Chemical Classification System, *ICD* International Classification of Diseases

The previous and only “Do not Do” recommendation stablished for headache in the local context, was kept and not only considered but it now approaches outpatients and emergency care. In this consensus other recommendations from international organizations have been considered and rephrased for the Spanish context, for instance don’t use opioid or butalbital treatment for migraine except as a last resort and don’t perform electroencephalography (EEG) for headaches [15]. Finally, three “Do not Do” recommendations were prioritized different from other previously stablished. These recommendations were related with preventive treatment drugs and its duration, and proper referral from primary care to specialized care.

Review of the medical records revealed that consensus “Do-not-Do” practices were implemented clinically in most of the hospitals participating in this study, although with varying frequency (Table 2). Practice NHC-02, involving withdrawal of preventive treatment before 15 days, was the most common, at 34.1% of the records reviewed. NHC-05, related to correct referral from the first level to neurology, had an occurrence of 4% and varied greatly between centers. Indicator data showed adequate use of diagnostic aids along with correct use of opiates, NSAIDs, and acetaminophen. Even thought that it is important to point out the high variability among hospitals in most of the “Do not do” recommendations predominantly in NHC-02, 04 and 05.

**Table 2:** Frequency with which the consensus “Do not Do” practices were implemented

Code	Numerator (N)	Denominator (N)	Indicator (%)	Range
NHC-01	53	3314	1.6	0–39.6
NHC-02	1136	3329	34.1	2.2–62.9
NHC-03	23	3329	0.7	0–8.3
NHC-04	694	3311	21.0	16.1–62.5
NHC-05	133	3326	4,0	0–58.2
NHC-06	635	3337	19,0	11–19.6
NHC-07	18	3361	0,5	0–8.8

Numerator: Number of patients with a certain diagnosis or condition who have received a treatment or procedure that they should not have received; cases of overuse defined by consensus of the expert group within a given time period. Denominator: The number of patients with a given diagnosis or condition who have been treated in the unit within a given time period

Recommendations related to diagnostic tests or procedures accumulated most of the avoidable expenses for the healthcare system, even though there is widely agreement on its low value in specific cases. Costs associated of ignoring this “Do not Do” vary from 88,15€ to 429,00€ each time it happens, depending on the average cost of the procedure or drug. NHC-02 which has been specially tailored and prioritized for Spanish hospitals has an individual direct cost per patient of 142,57 €. If we extrapolated this value to an estimated annual cost per 1000 patients of these seven low-value or undesirable practices, we could reach the amount of 203,520.47 euros (Table 3). If further action is performed to prevent NHC-02 and NHC-04 up to 75% of this expense could be saved.

**Table 3:** Estimated costs of ignoring “Do not Do” recommendations

Code	Average consultation cost (A)	Procedure/Drug (B)	Total direct cost (C = A + B)	Occurrence per 1000 patients (D)	Estimated cost (C × D) per 1000 patients
NHC-01	82,00 €	6,15 €	88,15 €	16	1.410,40 €
NHC-02	71,00 €	71,57 €	142,57 €	341	48.616,37 €
NHC-03	82,00 €	347,00 €	429,00 €	7	3.003,00 €
NHC-04	82,00 €	240,08 €	322,80 €	210	67.788,00 €
NHC-05	82,00 €	-	82,00 €	40	3.280,00 €
NHC-06	175,0 0€	240,08 €	415,80 €	190	79.002,00 €
NHC-07	82,00 €	2,14 €	84,14 €	5	420,70 €

A: Average consultation cost obtained from available information

B: Average cost of each diagnostic procedure or therapeutic guideline used

C: Total direct cost of low-value practices

D: Occurrence of “Do not Do” in 1000 patients

## Discussion

The concept of doing what is really needed rather than focusing on what is dispensable (Right Care) has gradually spread throughout health systems worldwide. The results of this study indicate that there is significant variability between centers in terms of the prescription and use of diagnostic tests that do not provide value or that may even be harmful to patients and emphasize the need for intervention to avoid these low-value practices.

The “Do not Do” recommendations for headache care developed by consensus in this study are supported by scientific evidence and experience in daily clinical practice. They address the entire care process, from correct referral from the first level to neurology consultation [16], to the overuse of neuroimaging without alarm criteria, defined in the consultation or emergency department [17], the inappropriate use of neurophysiological studies [18], the abuse of analgesic treatment [19, 20], the use of opioids for primary headaches [21], and the withdrawal of preventive medication [22]. Although these “Do not Do” recommendations are similar to some previously published by the American Headache Society [23] they have been elaborated in the context of the Spanish public health system, and are probably applicable to other national health systems. Three recommendations related with preventive treatment and a proper referral to specialized care could be added to low-value care literature.

Our results highlight that implementing these “Do not Do” practices not only jeopardize patient safety, but also endanger the integrity and maintenance of the National Health System. The main avoidable cost was related to non-recommended diagnostic tests, which may also be attributed to the practice of defensive medicine by practitioners, or to pressure from patients [24]. If we extrapolate these results to the whole of the health system in Spain, in terms of direct costs, this amounts to an annual figure of approximately 615 million euros, based on the last reported prevalence of 3,026,072 people diagnosed with migraine in 2015 [18].

The reduction of these practices with low diagnostic or therapeutic value or of practices that are harmful to patients has been achieved in some of the centers participating in this study (for example, the use of opiates for the treatment of primary headaches and the abuse of analgesics) by incorporating criteria for carrying out examinations or for prescribing medication. It is important to notice the high variability among the participating hospitals. This is due that some services, hospitals, or health areas in Spain can independently generate its own measures to reduce low value care. This can be for one instance the inability of prescribing opioids for headache or the active blocking of CT scans for headaches without alarm signs. This approach in implementation science has been described as PRECEDE, in which there are multiple factors, including knowledge, attitudes and beliefs (as predisposing factors), enabling factors, such as active decision support and work flow innovations in point-of-care tools as the electronic medical record and finally reinforcing factors (motivation to sustain behavior change) [24].

The literature reflects the outcome of interventions based on communication of the benefit of following these recommendations, acting on professional attitudes, knowledge, and behavior, or on changes in patient demands [25, 26]. For example, the results of the Choosing Wisely campaign are inconsistent; while significant reductions in some “Do not Do” practices have been achieved, there has been no change in the implementations of others by professionals [27, 28]. These and other findings on the level of awareness of these recommendations among professionals [29–31] suggest that more effort is needed to make these “Do not Do” recommendations known (also including campaigns directed at patients). They also emphasize that it is necessary not only to identify and measure low-value practices, but also to establish how to convey these results to the professionals and specialists involved in headache. These first two steps are essential for improving the quality of care provided and thereby reduce the cost of delivering such care.

There are also other possible factors that modify the compliment with “Do not Do” recommendations. Our study doesn’t approached other causes related to sociodemographic and personal characteristics of patient and provider as they have been discussed in other studies [32].

These recommendations pursue the objective of improving quality of healthcare and patient safety, and simultaneously lowering low value care. But “Do not Do” recommendations can’t effectively fulfill this aim by its own. It must be accompanied by other policies related to patient safety and quality of care [33]. These measures will backup physicians and allow them to provide better health care. Also, other techniques can help healthcare workers, for example watchful waiting [34]. A proper and trustful communication, between provider and patients have demonstrated in previous studies to improve outcomes and lowering the incidence of low value practice [25].

This study had some limitations. First, the quality of the information recorded in the clinical records may have led to under-identification of cases of overuse. Although an algorithm was defined with specification of diagnostic and therapeutic codes (numerators and denominators) to record the same data in all centers, possible variability in coding between centers cannot be ruled out. For ethical reasons, professionals could only review the clinical history of patients from their own center, and thus cross-validation of the overuse coding was not possible.

## Conclusion

In conclusion, the consensus “Do not Do” recommendations developed in this study are relevant to daily clinical practice. We found that the incidence of low-value practices varied depending on the hospital and the type of headache: there was greater variability in drug prescription, and more similar results in the overuse of neuroimaging. This study provided data on low-value practices in headache care, and also illustrated that a reduction of these low-value practices is both possible and desirable.

## Supplementary information

**Additional file 1: Supplemental file 1.** Do not Do recommendations proposed by the expert panel.

## Data Availability

The datasets used and/or analyzed during the current study are not available to ensure data privacy according the Spanish laws.

## Abbreviations

SIGN: Scottish Intercollegiate Guidelines Network
ICD: International Classification of Diseases
ATC: Anatomical Therapeutic Chemical Classification System
NSAIDs: Nonsteroidal anti-inflammatory drugs
