# Supplementary material for: Low-value care practice in headache: a Spanish mixed methods research study
Source: J Headache Pain. 2020 Jun 10;21(1):74. doi: 10.1186/s10194-020-01147-w (PMC7288523; doi:10.1186/s10194-020-01147-w)
Supplement: Supplementary file 1 — Additional file 1: Supplemental file 1. Do not Do recommendations proposed by the expert panel. [file 10194_2020_1147_MOESM1_ESM.docx]

Supplemental file 1. Do not Do recommendations proposed by the expert panel.

| **Code** | **Definition** |
| --- | --- |
| NHC – 01 | Do not recommend painkillers (NSAIDs, paracetamol, and others) more than 15 days per month in a primary headache that does not respond to treatment. |
| NHC – 02 | Do not withdraw preventive treatment in patients with primary headaches before 8 weeks if the efficacy and therapeutic dose is adequate. |
| NHC – 03 | Do not perform neurophysiological studies to establish the diagnosis of migraine or tension headache. |
| NHC – 04 | Do not perform neuroimaging studies on patients with tension headache, migraine without aura and with typical and episodic aura without alarm signs. |
| NHC – 05 | Do not refer patients from primary care with a diagnosis of primary headache without having prescribed symptomatic treatment with triptans or standard preventive treatment. |
| NHC – 06 | Do not perform complementary neuroimaging tests on an urgent basis on patients who do not meet the established alarm criteria. |
| NHC – 07 | Do not use opiates as a symptomatic treatment for primary headache. |
| NHC – 08 | Do not indicate alternative treatments (herbal medicines, nutraceuticals, homeopathy, and osteopathy) that do not have scientific evidence justifying the indication in migraine or other primary headaches. |
| NHC – 09 | Do not offer preventive treatment to patients who do not meet the criteria to be candidates for such therapy. |
| NHC – 10 | Do not recommend preventive treatments without considering the impact on the patient's comorbidity outcome. |
| NHC – 11 | Do not prescribe more than 3 months a preventive treatment without results in the patient with headache without modifying dose or without changing therapy. |
| NHC – 12 | Do not limit access to triptans in patients with primary headaches. |
| NHC – 13 | Do not make referrals from primary care of patients with headache as a guiding symptom to services other than Neurology. |
| NHC – 14 | Do not withdraw tolerated oral drugs at adequate doses before 4-6 weeks. |
| NHC – 15 | Patients attending the emergency services with headache, whether of primary or secondary cause, should not be considered a delaying emergency. |
| NHC – 16 | Patient's adherence to treatment should not be assumed. |
| NHC – 17 | Subjects with controlled and sporadic headaches should not be cited unnecessarily. |
